# Sensory innervation of the lumbar 5/6 intervertebral disk in mice

**DOI:** 10.3389/fneur.2023.1084209

**Published:** 2023-04-03

**Authors:** Lunhao Chen, Xuan Lu, Qianjun Jin, Zhihua Gao, Yue Wang

**Affiliations:** ^1^Spine Lab, Department of Orthopedic Surgery, The First Affiliated Hospital, Zhejiang University School of Medicine, Hangzhou, China; ^2^Department of Neurobiology and Department of Neurology of Second Affiliated Hospital, NHC and CAMS Key Laboratory of Medical Neurobiology, Zhejiang University School of Medicine, Hangzhou, China; ^3^The MOE Frontier Research Center of Brain and Brain-Machine Integration, Zhejiang University School of Brain Science and Brain Medicine, Hangzhou, China; ^4^Liangzhu Laboratory, Zhejiang University Medical Center, Hangzhou, China

**Keywords:** lumbar intervertebral disk, dorsal root ganglia (DRG), neuron, nerve terminal, retrogade tracing, neural pathways

## Abstract

**Introduction:**

Over the years, most back pain-related biological studies focused on the pathogenesis of disk degeneration. It is known that nerve distributions at the outer layer of the annulus fibrosus (AF) may be an important contributor to back pain symptoms. However, the types and origins of sensory nerve terminals in the mouse lumbar disks have not been widely studied. Using disk microinjection and nerve retrograde tracing methods, the current study aimed to characterize the nerve types and neuropathway of the lumbar 5/6 (L5/6) disk in mice.

**Methods:**

Using an anterior peritoneal approach, the L5/6 disk of adult C57BL/6 mice (males, 8–12 weeks) disk microinjection was performed. Fluorogold (FG) was injected into the L5/6 disk using the Hamilton syringe with a homemade glass needle driven by a pressure microinjector. The lumbar spine and bilateral thoracic 13 (Th13) to L6 DRGs were harvested at 10 days after injection. The number of FG^+^ neurons among different levels was counted and analyzed. Different nerve markers, including anti-neurofilament 160/200 (NF160/200), anti-calcitonin gene-related peptide (CGRP), anti-parvalbumin (PV), and anti-tyrosine hydroxylase (TH), were used to identify different types of nerve terminals in AF and their origins in DRG neurons.

**Results:**

There were at least three types of nerve terminals at the outer layer of L5/6 AF in mice, including NF160/200^+^ (indicating Aβ fibers), CGRP^+^ (Aδ and C fibers), and PV^+^ (proprioceptive fibers). No TH^+^ fibers (sympathetic nerve fibers and some C-low threshold mechanoreceptors) were noticed in either. Using retrograde tracing methods, we found that nerve terminals in the L5/6 disk were multi-segmentally from Th13-L6 DRGs, with L1 and L5 predominately. An immunofluorescence analysis revealed that FG^+^ neurons in DRGs were co-localized with NF160/200, CGRP, and PV, but not TH.

**Conclusion:**

Intervertebral disks were innervated by multiple types of nerve fibers in mice, including Aβ, Aδ, C, and proprioceptive fibers. No sympathetic nerve fibers were found in AF. The nerve network of the L5/6 disk in mice was multi-segmentally innervated by the Th13-L6 DRGs (mainly L1 and L5 DRGs). Our results may serve as a reference for preclinical studies of discogenic pain in mice.

## Introduction

Back pain is a common disorder in the lumbar spines of adults, affecting over 50% of people during their lifetimes (1). Although pathologies in a variety of anatomical structures, such as intervertebral disks, facet joints, myofascial tissues, and sacroiliac joints, may lead to back pain (2), intervertebral disk pathology is thought to be a significant contributor to back pain, termed discogenic pain (3). Identifying the innervation patterns of nerve terminals in the disk may serve as an important reference in studying discogenic pain.

The disk is referred to as the largest avascular tissue of the body, which comprises an outer ring of collagen-rich annulus fibrosus (AF) and proteoglycan-rich gelatinous nucleus pulposus (NP) in the center, providing both the mechanical support for compressive forces and the flexibility necessary for the movements (4). Previous studies indicated that the outer one-third layers of the normal AF are innervated by different types of sensory nerve terminals, including both myelinated and unmyelinated fibers (5, 6). Using immunohistochemical staining and structure analysis, peptidergic nerve fibers, containing calcitonin gene-related peptide (CGRP) or substance P (SP), and mechanoreceptors, containing neurofilament heavy polypeptide (NEFH), were found in disks and suggested to sense nociceptive and mechanical stimuli (7, 8). After disk injury and degeneration, those nerve terminals can sprout and extend deeper, even up to the inner two-thirds of AF and NP, which was considered as a possible pathomechanism of discogenic pain (9).

Moreover, during disk degeneration, AF and NP cells upregulate the expression of proinflammatory cytokines, including interleukins, tumor necrosis factors (TNFs), and metalloproteases (MMPs), which interact with corresponding receptors on nerve terminals to promote nerve ingrowth, and therefore facilitate ectopic action potentials and discogenic pain (10–12). Using the mouse model of disk degeneration, disk puncture induced progressive disk degeneration, along with the increased expression of colony-stimulating factor 1 (CSF1) in dorsal root ganglion (DRG) and activated microglia in the lumbar spinal dorsal horn (13), suggesting that neural plasticity was involved in back pain.

Anatomically, the intervertebral disk is assumed to receive afferent fibers principally from DRG neurons of the same segment. Yet, the lower lumbar intervertebral disks are multi-segmentally innervated by DRG neurons, whose nerve terminals enter the paravertebral sympathetic trunks and ascend to upper-level DRGs (2, 14, 15). Local anesthetic blocks to sympathetic ganglia at the L2 level alleviated discogenic pain in patients (16, 17), and experimental studies in rats demonstrated a raised pain threshold after sympathectomy (18). However, due to the adjacent distance between the disk and corresponding DRG, proinflammatory cytokines are presumed to affect the adjacent DRG neurons, largely complicating the origin of discogenic pain.

Previous studies mainly used rats as experimental models to distinguish the distribution and innervation patterns of the disk (2, 5, 14, 19–22). However, the types and origins of sensory nerve terminals in the lower lumbar disks in mice remain elusive. Moreover, transgenic tools and genetic manipulations are limited in rats, conferring a disadvantage in uncovering the mechanistic clues of the disease. Using disk microinjection and nerve retrograde tracing methods, the present study was conducted to get a better understanding of the nerve types and neural pathways of the lower lumbar disk in mice.

## Materials and methods

### Animals

Adult C57BL/6J mice (males, 8–12 weeks) were used in the present study. All animals were housed in the Lab Animal Center at Zhejiang University, and the group was housed on a 12-h light/dark cycle with water and food available. The use and care of animals in all experiments followed the guidelines of The Tab of Animal Experimental Ethical Inspection of the First Affiliated Hospital, College of Medicine, Zhejiang University, Hangzhou, China (No. 2017054). For each experiment, at least three mice per group were used.

### Disk injection

**Figure 1:**
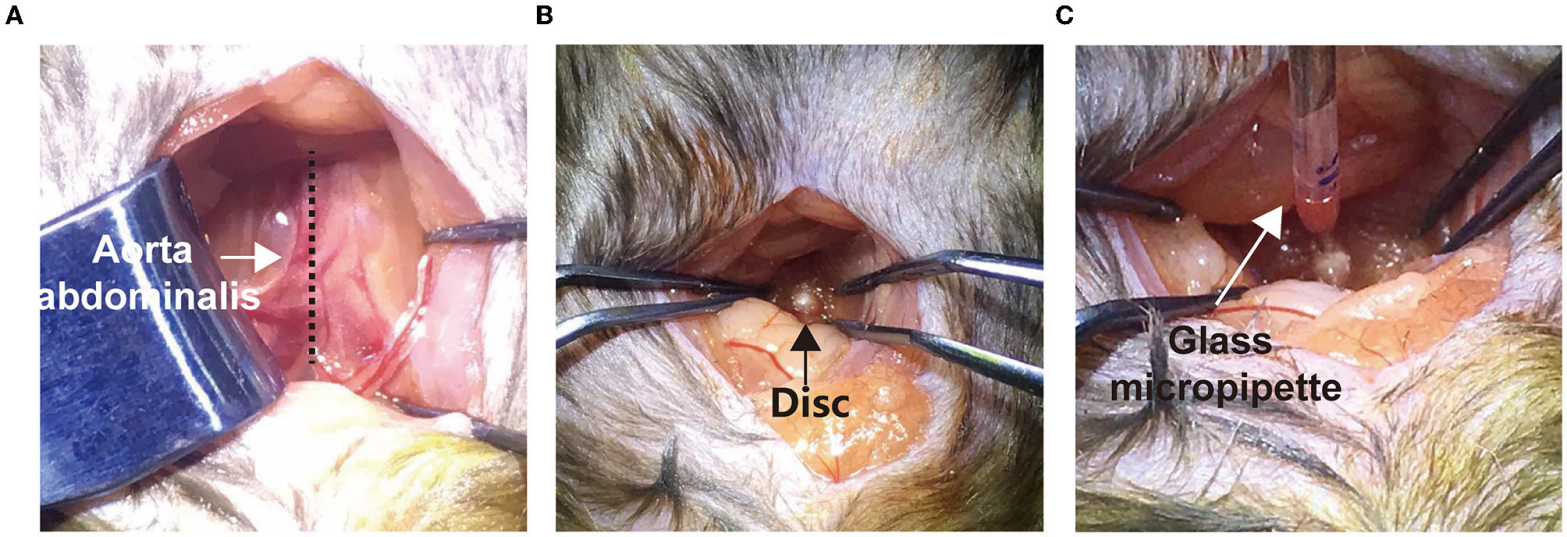
Surgical procedure of disk microinjection through a transabdominal approach. **(A)** A middle longitudinal incision was made to expose the aorta abdominalis (arrow). The gut and caecum were gently pushed aside to expose the psoas muscles (dotted line). **(B)** Using smooth clamps, the L5/6 disk was further exposed (black arrow). **(C)** Using a glass micropipette and a nano-electronic microinjection pump, FG was slowly injected into the disk.

To minimize the disk injury, we connected the 10 μL Hamilton syringe (Cat# 701N) with a glass needle (diameter ≈ 20 μm) before disk injection. Before the experiments, the mice were fasted for 8 h prior to disk injection. After being anesthetized with sodium pentobarbital (100 mg/kg) intraperitoneally, the mice were placed and fixed in a supine position on a warming pad. Under the sterile surgical condition, a 1–1.5 cm midline longitudinal incision in the lower abdomen region was made in mice. Under a stereoscopic microscope (SZX7, Olympus), the gut, connective tissues, vessels, and psoas major muscles were gently retracted to expose the ventral aspect of the L5/6 and L6/sacral 1 (S1) intervertebral disk (Figures 1A, B). The weight/volume (w/v) ratio of 1% FG was infused using the homemade Hamilton syringe with a glass needle and driven by a pressure microinjector (KD Scientific) at a rate over at least 5 min (Figure 1C). The injection depth was 1 mm, and the total injection volume was 100 nL, which avoided breaking through the posterior annulus. To allow diffusion and reduce backflow, the needle remained in the disk for 5 min. The incision was then closed with 5–0 silk sutures.

### Disk and DRG dissection

Ten days after the surgery, mice were anesthetized and perfused with saline and subsequently 4% paraformaldehyde (PFA). The lumbar spine and DRGs were dissected and post-fixed in 4% PFA for at least 6 h. After decalcifying in 10% ethylene diamine tetra-acetic acid (EDTA) for 14 days and dehydrating in 30% sucrose for 2 days, the lumbar spine was sagittally sectioned with 10-μm thickness. DRGs were sectioned in transverse with 15-μm thickness.

### Counting of FG+ neurons

All DRG sections from bilateral Th13-L6 levels were used in counting FG^+^ neurons. FG was visualized as granular particles within neurons. The FG-positive neurons were defined as the white-blue rounded cells under a fluorescence microscope (BX53, Olympus), whose fluorescence intensity was much higher than the background. To avoid double counting of FG-labeled neurons, the second one in the adjacent section was ignored if FG-labeled neurons appeared at the same location in two adjacent sections. The number of FG^+^ neurons was mounted by each DRG level.

### Hematoxylin and Eosin (HE) staining

After rehydrated in alcohol, the sagittal lumbar spine sections were washed with distilled water. The sections were stained with hematoxylin for 1 min and differentiated with acid alcohol. After that, sections were stained with eosin for 1 min and dehydrated in ascending alcohol. Rinse the staining with xylene to make it transparent and mount. The figures were acquired using a microscope (BX53, Olympus).

### Immunofluorescence and immunohistochemical analysis

To label different types of nerve terminals in disks, the midsagittal sections of the lumbar spine were stained using a specific rabbit (Cat# K401011) and mouse (Cat# 400611) IHC Kit (DAKO) according to the manufacturer's instruction. Shortly, sections were antigen-retrieved in citrate buffer (10 mM sodium citrate, 0.05% Tween-20, pH 6.0) at 95°C for 20 min and permeabilized with 0.5% Triton X-100 for 10 min at room temperature. After being blocked with 10% (wt/vol) BSA, sections were incubated overnight at 4 °C with the following primary antibodies: mouse anti-NF160/200 (1:2000, Cat# n2912, Sigma-Aldrich), mouse anti-CGRP (1:1000, Cat# sc-57053, Santa Cruz Biotechnology), mouse anti-parvalbumin (PV, 1:1000, Cat# P3088, Sigma-Aldrich), rabbit anti-tyrosine hydroxylase (TH, 1:1000, Cat# AB152, Millipore), and rabbit anti-flurogold (FG, 1:1000, Cat# 52-9600, Fluorochrome). To label disk nerve terminals, sections were washed in TBS with 0.5% tween (TBS-T) and incubated with HRP-conjugated secondary antibodies (1:1000) for 1 h at room temperature. Images were acquired using a microscope (BX53, Olympus). For DRG and spinal cord slides, sections were incubated with fluorophore-conjugated secondary antibodies for 2 h at room temperature. 4′,6-diamidino-2-phenylindole (DAPI, C1005, Beyotime, China) was used to label cell nuclei. Images were acquired using a confocal microscope (FV1200, Olympus).

### Statistical analysis

The number of FG^+^ neurons from Th13 to L6 DRGs was counted under a fluorescent microscope (BX53, Olympus, Japan). The percentage of DRG neurons per lumbar level was calculated as the number of DRG neurons in each lumbar level divided by the total number of FG^+^ neurons. Data are presented as means ± standard errors of the means (SEM). One-way ANOVA followed by Bonferroni's *post hoc* tests was used to compare the distribution of FG^+^ neurons among DRG levels. Significance was considered with a *p*-value of < 0.05.

## Results

### Segmental distribution of FG-labeled DRG neurons in mice

**Figure 2:**
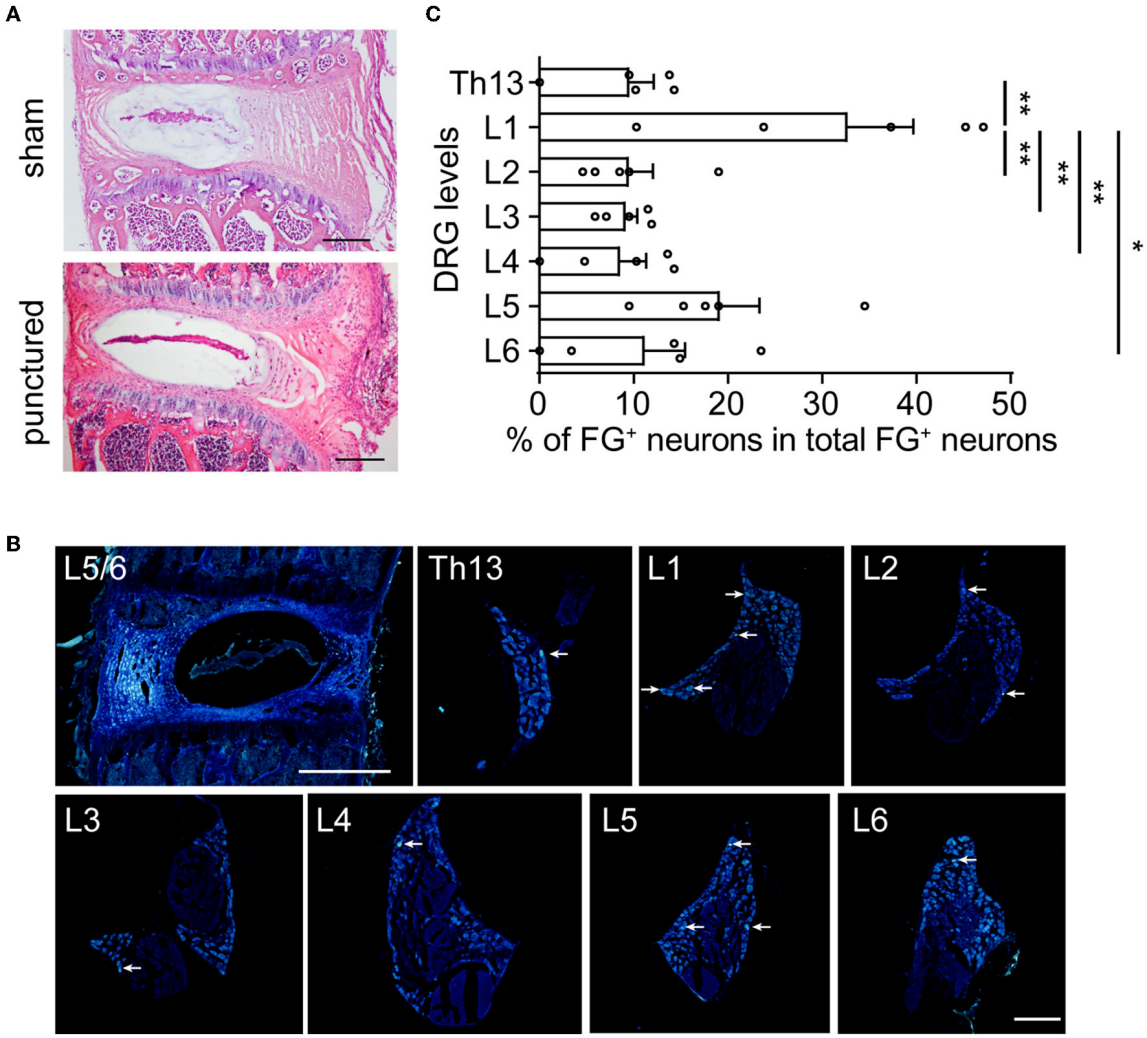
In mice, the L5/6 disk was multi-segmentally innervated by Th13-L6 DRGs (mainly L1 and L5 DRGs). **(A)** HE staining of L5/6 disks at 10 days after sham surgery (upper) or glass needle puncture (bottom). Scale bar: 200 μm. **(B)** Representative images of the disk and DRG after FG injection. Arrows indicate FG^+^ DRG neurons. **(C)** The distribution of FG^+^ DRG neurons of different spinal levels (*n* = 5 mice). The percentage represents the number of FG^+^ neurons for each level of DRG to total FG^+^ neurons from Th13 to L6 (*n* = 5 mice). Scale bars: 200 μm for DRG and 500 μm for disk. Values are means ± SEM. *p < 0.05 and **p < 0.01, one-way ANOVA followed by Bonferroni's *post-hoc* tests.

To label DRGs that innervate the L5/6 disk, the disk with FG injection and bilateral lumbar DRGs were acquired and observed under fluorescent microscopy. Compared with the disk that underwent sham surgery, HE staining demonstrated that the glass needle puncture did not induce disk degeneration 10 days after surgery (Figure 2A). As shown in Figure 2B, FG was visualized as white-blue colors. First, we recognized that FG was dispersed but limited in the L5/6 disk, without penetrating the vertebral bodies (Figure 2B). Although FG^+^ neurons were present from Th13 through L6 DRGs (Figure 2B, Table 1), FG^+^ neurons were most frequently observed in L1 DRGs (27.9% of all labeled neurons), followed by L5 DRGs (22.1%) (Figure 2C). Only 18.8% of FG^+^ neurons were identified in L5 DRG in mice. About 10% of FG^+^ neurons were found in Th13, L2, L3, L4, or L6 DRGs, respectively (Figure 2C). These data suggested that sensory fibers in the L5/6 disk in mice were segmentally innervated by Th13-L6 DRGs.

**Table 1:** Number of FG^+^ neurons in DRGs.

**Mouse**	**Th13**	**L1**	**L2**	**L3**	**L4**	**L5**	**L6**	**Total**
1	6	19	8	3	2	4	0	42
2	2	5	2	2	3	4	3	21
3	6	22	5	7	8	9	2	59
4	12	9	4	10	9	30	13	87
5	0	8	1	1	0	3	4	17
Total	26	63	20	23	22	50	22	226

### Types and distributions of nerve terminals in the intervertebral disks in mice

**Figure 3:**
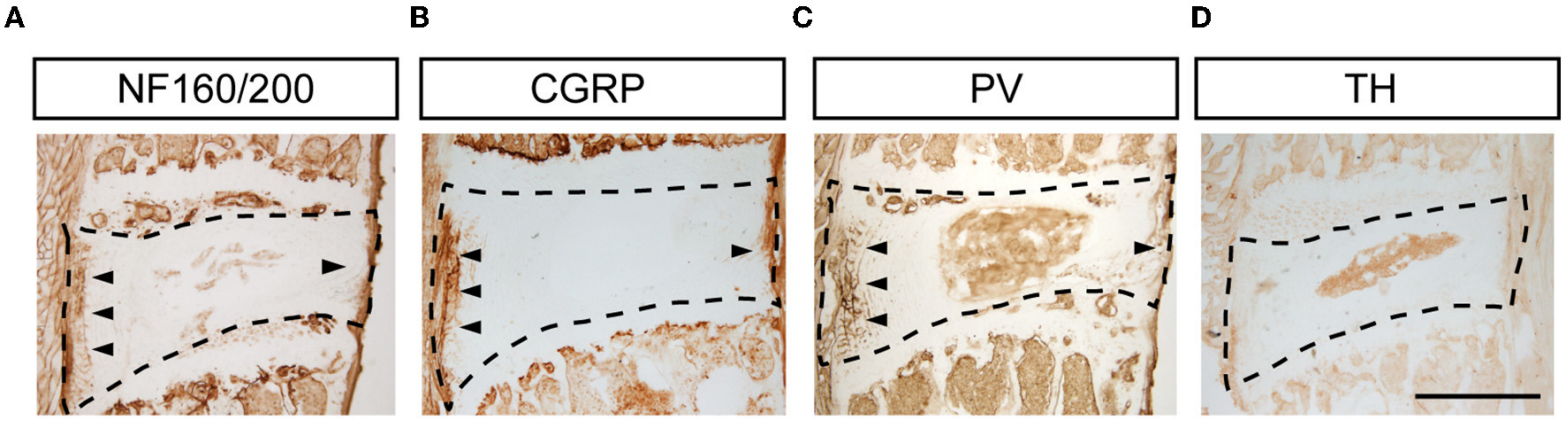
Types of nerve terminals in L5/6 disk in mice. Representative images of NF160/200^+^ **(A)**, CGRP^+^ **(B)**, PV^+^ **(C)**, and TH^+^ **(D)** staining in disks. Scale bar: 500 μm.

**Figure 4:**
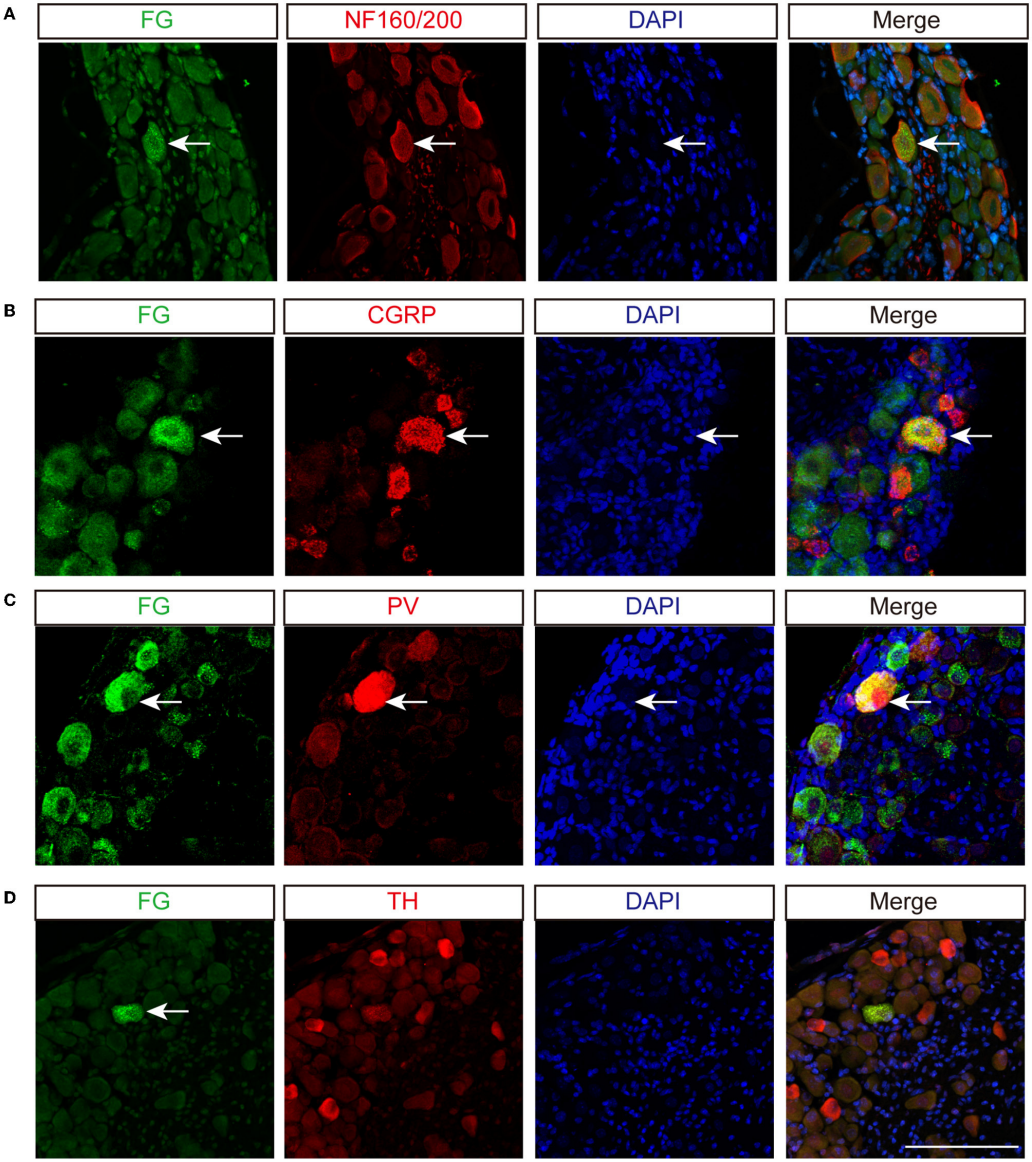
Sensory innervation of L5/6 disk in mice. Immunofluorescence labeling of FG with NF160/200 **(A)**, CGRP **(B)**, PV **(C)**, and TH **(D)** in DRG. Arrows indicate co-labeled neurons. Scale bar: 100 μm.

To identify types of DRG neurons whose nerve fibers project to the disk, we co-labeled FG with different neuronal markers, which are thought to cover major portions of sensory neurons (23). Consistent with previous studies, CGRP, the marker of small peptidergic neurons with Aδ- or C-type fibers, was observed in the outer layer of the AF (Figure 3A). NF160/200, the marker of large peptidergic neurons with Aβ-type fibers, was also identified in the superficial region of AF (Figure 3B). Intriguingly, we found that PV^+^ fibers (Figure 3C), the marker of proprioceptors, were densely aligned within AF, demonstrating that intervertebral disks may sense proprioception, such as position and movement. However, we were unable to identify TH^+^ fibers (Figure 3D), which indicated unmyelinated C-low threshold mechanoreceptors (C-LTMRs) and sympathetic nerve fibers, in the AF. Immunofluorescent staining of the DRG sections demonstrated that FG in DRGs was partially co-localized with NF160/200, CGRP, and PV, but not TH in mice (Figure 4). Together, our data demonstrated that disks were innervated with different types of sensory nerve fibers, which were susceptible to sensing mechanical and chemical stimuli in the internal environment.

## Discussion

Although there have been several reports about the sensory innervation of lumbar disks in rats, our study describes the distribution patterns of sensory nerves in the mouse lumbar disk and their origins in DRG neurons. Consistent with rats, the sensory fibers of the L5/6 disk were shown to be derived from DRGs at Th13 through L6. Moreover, immunohistochemical staining demonstrated that there were diverse types of nerve terminals, including NF160/200, CGRP, and PV, in the outer layers of AF, suggesting that such neural structures of disks may be susceptible to sense stimuli by mechanical or chemical mediators after herniation and degeneration, and further contributed to discogenic pain.

There have been efforts trying to identify the distribution patterns of disk innervations in rats and humans. Anatomically, the lumbar disk is innervated by sinuvertebral nerves consisting of spinal sensory fibers from the adjacent DRG and postganglionic sympathetic fibers, which demonstrates that the lumbar disk is innervated by DRG at corresponding levels and upper DRGs (24). However, using retrograde tracing methods in rats, the ventral portion of the L5–L6 disk was innervated predominantly by L1 and L2 DRGs (14), whereas the dorsal portion of the L5/6 disk was innervated extensively by L1–L6 DRGs (2). To avoid FG leakage, we used the microinjection pump with a tiny tip to deliver FG. Consistent with previous studies, our findings support the multi-segmental innervation patterns of L5/6 disk in mice: the origin of nerves was thought to enter the paravertebral sympathetic trunks and ascended to L1 and L2 DRGs, whereas some fibers passed through the sinuvertebral nerves and reached L3–L6 DRGs (15). Indeed, the human L4/5 disk, which is related to the L5/6 disk in rodents, may be innervated predominantly by L1 DRG ventrally and by L2 DRG dorsally (25). As L1 and L2 DRGs innervate the inguinal region, it may explain why patients with lower lumbar intervertebral disk lesions are suffered from inguinal pain. Moreover, blocking L2 DRG alleviated some but not all of the low back pain, inguinal pain, and buttock pain in patients (17).

Nerve terminals in the outer layers of disks, which penetrated deeper after disk degeneration or herniation, provide a morphologic basis for discogenic pain (5). Disk injury and degeneration result in the upregulation of pain-related molecules, including CGRP and Substance P in DRGs, which are known to irritate the spinal nerve roots and probably also the nerve terminals to promote discogenic pain. Other than CGRP, the present study revealed that there were other types of nerve terminals in the outer layers of disks, including PV and NF160/200. Peripheral pain perception is thought to be mediated primarily by nociceptive C-fiber neurons and thinly myelinated Aδ fibers (soma diameter < 30 μm) (26). Growing evidence indicated that CGRP, one of the inflammatory mediators and the marker for nociceptive C fibers, was upregulated in DRG neurons after disk degeneration, as well as in aging-induced degenerated disks (5). These findings suggest that increased innervation by C-fibers is associated with discogenic pain.

Intriguingly, we observed the density of NF160/200- and PV-positive nerve fibers in AF. As NF160/200 and PV are regarded as markers of myelinated nerve fibers and mechanoreceptors that are derived from large-sized DRG neurons, they are responsible for transducing vibratory and position sensation (27). It is noteworthy that myelinated fibers have been implicated in neuropathic pain, largely through the activation of sodium channels (27). Blocking A-fiber neuron activity alleviated neuropathic pain (28). Intriguingly, our recent study demonstrated that mTOR activation in large-sized DRG neurons promoted nociceptor excitability and neuropathic pain (29). PV is a calcium-binding protein that presents in proprioceptors and low-threshold mechanoreceptors (30, 31). The mean PV expression was 25% of L4 or L5 DRG neurons, and this was unchanged 2 weeks after peripheral nerve injury (30). More recently, hyperpolarization-activated cyclic nucleotide-gated channel (HCN) was found on PV^+^ neurons, and the excitability of proprioceptive afferents may contribute to the sensorimotor properties (32). Although the presence of mechanoreceptors was more frequent in diseased disk patients with pain (7, 33), further studies are warranted to define the functional role of myelinated fibers in discogenic pain. It should be noted that, other than four markers used in the present study, there are more nerve subtypes, including MRGPRD (MAS-related GPR family member D), P2X3 (purinergic receptor P2X, ligand-gated ion channel, 3), and TRP (transient receptor potential) family, have been proved to play a role in pain development and maintenance (34–36). Further studies are needed to distinguish these nerve subtypes in the disks and examine their roles in discogenic pain.

The innervation of the disk is the structural base for inducing discogenic pain. Growing evidence suggested that disk degeneration can induce increased innervation of CGRP protein gene product 9.5 (PGP9.5, a broad neuronal marker) fibers (37). Although our findings and previous findings indicated that the number of DRG neurons innervated disk is limited (2, 14), disk degeneration has been proven to induce the upregulation of neurotrophins and neuropeptides, such as nerve growth factor (NGF), CSF1, CGRP, and NPY in both disks and DRG neurons, and activate microglia and astrocytes in the spinal dorsal horn (5, 13, 38). These findings indicated that disk degeneration upregulates various pain-related neuropeptides in DRG neurons as well as immune responses in the spinal dorsal horn. Moreover, these changes contribute to neuronal plasticity, which may enhance nociceptive signaling and lead to discogenic pain (3, 5, 26, 39).

There are some limitations in the present study. First, the distribution and innervation patterns of the ventral and dorsal portions of the lumbar disk in mice should be separately studied. However, due to the limited efficiency of FG retrograde tracing (2), the percentage of FG^+^ neurons to a total number of DRG was relatively low. Although myelinated fibers have been found in healthy disks, the changes in distribution patterns after disk degeneration and herniation remain elusive. Moreover, the quantification of subtype DRG neurons that innervate disks is needed. In addition, it is important to elucidate the roles of myelinated fibers in discogenic pain. Further electrophysiologic, optogenetics, chemical genetics, and clinical studies are required to elucidate the functional implications of this study. Although there are differences between humans and rodent nociceptors in pain sensitization, rodents serve as important models for back pain research. Further studies, such as single-cell RNA sequencing, are warranted to investigate similarities and differences between humans and rodents regarding cellular and molecular aspects of sensory neurobiology (40).

In conclusion, our current study demonstrated that sensory neurons, which multi-segmentally innervated the L5/6 disk, existed in Th13 to L6 DRGs in mice. Such an innervation pattern may explain why patients with lower disk degeneration experience inguinal and anterior thigh pain corresponding to the upper levels (L1 and L2) of DRGs. Using retrograde tracing methods, we also observed different types of nerve terminals derived from DRG neurons in mouse disks, including CGRP, NF160/200, and PV. The densely innervated structures of the lumbar disk may be responsible for sensing internal mechanical and chemical stimuli, and further contribute to discogenic pain.

## Data availability statement

The raw data supporting the conclusions of this article will be made available by the authors, without undue reservation.

## Ethics statement

The animal study was reviewed and approved by the Tab of Animal Experimental Ethical Inspection of the First Affiliated Hospital, College of Medicine, Zhejiang University.

## Author contributions

LC, XL, YW, and ZG designed experiments and wrote the manuscript. LC, XL, and QJ performed experiments and analyzed the data. YW supervised the project.
